# Association of Asthma With Patients Diagnosed With Metabolic Syndrome: A Cohort Study in a Tertiary Care Hospital

**DOI:** 10.7759/cureus.47558

**Published:** 2023-10-24

**Authors:** Jishna G, Elen Abraham, Ghanshyam Verma, Leny T Mathew, Sourya Acharya, Sunil Kumar, Keyur Saboo, Rinkle Gemnani

**Affiliations:** 1 Department of Respiratory Medicine, Sree Balaji Medical College and Hospital, Chennai, IND; 2 Department of Neurology, Mar Baselios Medical Mission Hospital, Ernakulam, IND; 3 Department of Medicine, Jawaharlal Nehru Medical College, Datta Meghe Institute of Higher Education and Research, Wardha, IND

**Keywords:** obesity, spirometric values, pulmonary function test, metabolic syndrome, control, bronchial asthma

## Abstract

Introduction

Asthma is defined as a chronic inflammatory airway disease. The prevalence of both asthma and obesity has been rising simultaneously, demonstrating a parallel trend. Obesity is a significant factor in metabolic syndrome, and numerous studies have indicated a connection between metabolic syndrome and bronchial asthma.

Aims and objectives

The aim of this paper is to evaluate the association of asthma with patients diagnosed with metabolic syndrome. The main objectives were to analyze the clinical profile and spirometric indices in patients with metabolic syndrome and to assess asthmatic patients among them with spirometry and clinical parameters at a tertiary care hospital in Chennai.

Materials and methods

This hospital-based cohort study was conducted on 73 patients attending the outpatient department who had a known case of metabolic syndrome and were evaluated for asthma through history, physical examination, and a pulmonary function test. A history of cough, expectoration, shortness of breath, wheezing, chest tightness, allergy, seasonal variation, and smoking habits was asked, and a thorough physical examination was performed. Bronchial asthma was confirmed with airflow reversibility by spirometry as per the Global Initiative for Asthma Guidelines. Metabolic and spirometry parameters were examined, such as body mass index (BMI), waist circumference, waist-hip ratio, fasting blood sugar (FBS), postprandial blood sugar, hemoglobin A1C (HbA1C), serum insulin, lipid profile, C-reactive protein (CRP), forced expiratory volume in the first second (FEV1), forced vital capacity (FVC), and FEV1/FVC pre- and post-reversibility (baseline vs. six months).

Results and discussion

The average BMI of all participants was 29.6511 ±2.64564. The waist-hip ratio was 0.5512 ±0.43855, which decreased during the follow-ups, demonstrating a decline in the risk of obesity in study participants. The level of HbA1C showed a drop from 6.1% to 5.9% at the first follow-up. This exhibited a further reduction at the six-month follow-up in addition to a positive reflection in insulin sensitivity, indicating successful control of diabetes among study participants. It was discovered that this was statistically significant (p<0.001). At the third and sixth months of follow-up, the FEV1/FVC ratio increased by 38% and 37%, respectively, when metabolic syndrome was under control. The results show that controlling diabetes, hypertension, obesity, and triglyceride values improved asthmatic symptoms, and this was determined to be statistically significant (p<0.001).

Conclusion

The results of the current study demonstrated that the regulation and maintenance of metabolic parameters such as BMI, diabetes, hyperlipidemia, and hypertension aid in improving asthma control.

## Introduction

Syndrome X, as described by G.M. Reaven in the late 1980s, seems to have marked the beginning of the modern period of what we now refer to as the "metabolic syndrome" or the "insulin resistance syndrome" [1]. Metabolic syndrome is a collection of connected risk factors with metabolic origins that can result in a variety of systemic diseases in people. Metabolic syndrome was defined according to the WHO criteria consisting of hyperinsulinemia or hyperglycemia (fasting glucose ≥110 mg/dl) in addition to at least two of the following: waist girth ≥94 cm, dyslipidemia (triglycerides ≥150 mg/dl or high-density lipoprotein (HDL) cholesterol <40 mg/dl), or blood pressure (BP) ≥140/90 mmHg or taking BP medication [1]. The metabolic risk factors are a result of a different set of circumstances known as the underlying risk factors. In recent years, several expert groups have made an effort to develop straightforward diagnostic standards that can be applied in clinical settings to identify patients who exhibit numerous symptoms of metabolic syndrome. The particular components of these criteria have varied slightly, but overall, they combine underlying factors as well as metabolic risk factors [2].

Although several metabolic disorders have been found to be independently connected to asthma, metabolic dysfunction is identified in asthma as associated with obesity [3]. The concepts through which the pathophysiology of many lung disorders is understood are changing, and as a result of this, there is advancement in our understanding of metabolic mechanisms [4].

The state of systemic inflammation carried by metabolic syndrome may explain the onset and severity of asthma. This is a relatively uncharted region that may create new scenarios for the strategic approach and the diagnostic algorithm, with a more thorough evaluation of the condition [5,6].

Asthma symptoms can be triggered by stimuli like exercise, allergens, changes in the weather, or viral respiratory infections due to the hyperresponsiveness of the airways in the disease [7]. Asthma symptoms include persistent coughing, episodes of wheezing, shortness of breath, and tightness in the chest [8,9,10,11]. These symptoms are frequently worse at night or after exercising.

Asthma is a diverse disease that can be caused by various underlying disease processes. 'Asthma phenotypes' are identifiable groups of randomized demographic, clinical, and/or pathophysiology traits [12,13,14]. There are several different clinical manifestations of asthma. Some of the common manifestations include allergic asthma, non-allergic asthma, adult-onset (late-onset) asthma, asthma with persistent airflow limitation, and asthma with obesity. Sometimes obese asthmatic patients exhibit severe asthma symptoms but only mild eosinophilic inflammation. In addition to higher triglycerides and lower HDL cholesterol, the metabolic syndrome also includes high blood pressure, diabetes, or excessive glucose levels. Although the underlying mechanisms are still not completely understood, metabolic disruption is identified in asthma associated with obesity.

It appears that the presence of three of the following symptoms is necessary for the diagnosis of metabolic syndrome: alterations in fasting glucose metabolism, dyslipidemia, hypertension, and obesity. Additionally, metabolic syndrome is directly related to an increased risk of atherosclerosis and coronary heart disease [15,16]. Additional typical clinical indicators of metabolic syndrome include liver failure, sleep apnea, and chronic inflammatory conditions like asthma. Although the relationship between asthma and metabolic syndrome has been looked at in several studies, to our knowledge, no meta-analysis has looked into the relationship [11,17].

A study of the so-called "thin fat" people, who have a normal body mass index (BMI) but display changes that are indicative of metabolic syndrome, is necessary to establish the relative contributions of metabolic syndrome and obesity to the increased risk of developing asthma. These investigations are ongoing. Numerous cell types, including vascular endothelial cells, renal epithelial cells, and glandular epithelium, have altered activities in the metabolic syndrome. It is unknown but extremely possible that people with metabolic syndrome have altered airway epithelium or sub-epithelial function [18,19].

In a Korean health and genome study, 9,942 adults between the ages of 40 and 69 were examined for their associations with asthma-like symptoms and metabolic syndrome [20]. When compared to people without metabolic syndrome, subjects with metabolic syndrome had a considerably higher prevalence of wheezing, resting dyspnea, and post-exercise dyspnea, which are asthma-like symptoms. The metabolic syndrome criteria, abdominal obesity, and high blood pressure were all substantially linked to the asthma-like syndrome. One of the largest population-based studies to demonstrate the link between metabolic syndrome and asthma was the Incidence of Asthma and Metabolic Syndrome study [3].

Furthermore, a few researchers found links between asthma and the three metabolic syndrome symptoms (obesity, hypertension, and increased fasting glucose/diabetes in unadjusted models) [21]. However, when the models were adjusted for BMI, it was discovered that BMI, not metabolic syndrome, is significantly related to asthma in females. It is clear from the studies stated above that the key risk factors linking metabolic syndrome and asthma are abdominal obesity, insulin resistance (elevated glucose), and hypertension [21].

Overall, based on these risk variables, there are several possible explanations for the association between metabolic syndrome and asthma. It may be brought on by mechanical reasons, genetic and epigenetic factors, inflammatory factors, mitochondrial dysfunction, hormonal factors, and other co-morbidity effects, among others [22].

Thus, the current study's objective was to assess the distribution and associations of asthma in patients with metabolic syndrome, as well as the relationship between changes in systemic metabolism and its correlation with asthma.

## Materials and methods

This cohort study was carried out among the outpatients attending the Department of Pulmonary Medicine and Internal Medicine at Sree Balaji Medical College and Hospital, Chennai, from March 2021 to November 2022. The nature and purpose of the study were explained to the Institutional Review Board, following which ethical clearance was obtained to conduct the study (Institutional Human Ethical Committee of Sree Balaji Medical College and Hospital, Ref No. 002/SBMC/IHEC/2021/1595).

The inclusion criteria were age >18 years and patients who were known cases of metabolic syndrome attending the outpatient department of internal medicine and pulmonary medicine. Exclusion criteria were age >80 years, chronic smokers, patients with chronic respiratory diseases such as interstitial lung disease (ILD) and bronchiectasis, and patients with active infection. Participants from all socioeconomic backgrounds were present in the current study. Each participant's occupation was recorded, and it was discovered that participants from low social backgrounds, such as cooks, farmers, and electricians, and participants from middle and upper social backgrounds, such as engineers, teachers, and businessmen, were represented in the study.

Training and calibration

Before the commencement of the study, the investigator was trained and calibrated to ensure uniform recording of values from the procedures. Training exercises were carried out under the guidance of an experienced clinician. Following the training and calibration exercises, the inter-examiner reproducibility between the investigator and the calibrator for the procedures was recorded for a group of 10 adults, and the inter-examiner reproducibility was found to be 92%. Calibration of the equipment was done regularly on the day of testing.

Sample size

Keeping the power of the study at 95% and the probability of type I error (α) at 5%, the sample size for the main study was calculated based on the prevalence value obtained from previous literature: n= z2 X ^P (1- ^P) / E2 Z= z score. Confidence interval: 95%; E: margin of error: 5%; population proportion for asthma with metabolic syndrome in India: 5%; P = population proportion. Hence, the final sample size was 73.

A history of cough, expectoration, shortness of breath, wheezing, chest tightness, allergy, seasonal variation, and smoking habits was obtained, and a thorough physical examination was done. Bronchial asthma was confirmed with airflow reversibility by spirometry. Metabolic and spirometry parameters were examined, such as body mass index (BMI), waist circumference, waist-hip ratio, fasting blood sugar (FBS), postprandial blood sugar (PPBS), hemoglobin A1C (HbA1C), serum insulin, lipid profile, C-reactive protein (CRP), forced expiratory volume in the first second (FEV1), forced vital capacity (FVC), and FEV1/FVC pre- and post-reversibility (baseline vs. six months). Worsening of symptoms in terms of cough, expectoration, shortness of breath, wheezing, and chest tightness was considered. Values were recorded and noted in a Microsoft Excel sheet (Microsoft Corp., Redmond, WA), decoded, and coded against the standard values. The baseline values of spirometry and metabolic parameters were assessed and compared on follow-up after six months. The association and distribution of asthma in metabolic syndrome were assessed in the study population, and the correlation between control metabolic parameters and their effect on asthma was evaluated with spirometry and clinically on follow-up.

Method of spirometry

The spirometry was carried out as a planned procedure as per the American Thoracic Society/European Respiratory Society (ATS/ERS) guidelines using an EasyOnePro, EOP-600448 machine (ndd Medical Technologies, Andover, MA). Positive bronchodilator (BD) responsiveness (reversibility test): increase in FEV1 of > 12% and > 200 mL The three best performances were selected. The average value of the three was taken as the final value of the patient.

The primary outcome of the study was to evaluate the association between asthma and patients diagnosed with metabolic syndrome. The secondary outcome was to assess asthmatic patients with spirometry, clinical parameters, and the association with metabolic syndrome.

Statistical analysis

Collected data were entered in a Microsoft Excel sheet and analyzed by descriptive and inferential statistical methods using IBM Statistical Package for the Social Sciences (SPSS) software version 22.0 (IBM Corp., Armonk, NY) and Prism version 6.0 (GraphPad Software, La Jolla, CA). The Pearson coefficient was applied to the correlation between pre- and post-effect analyses. Descriptive methods such as frequency and percentage were obtained to summarize the data for categorical types of parameters such as age, sex, pulmonary function test (PFT) findings, etc. The level of statistical significance was <0.05.

## Results

Out of a total of 73 study participants, 29 (39.72%) were between the ages of 50 and 59, with females being 40 (54.8%) and males 33 (45.2%). Among them, 41 patients were asthmatics, with 27 (36%) being females and 14 (19%) being males. The presence of cough was among 29 (39.7%) participants, with the absence of expectoration in 53 (72.6%). The absence of shortness of breath was among 65 (89%) and present among 8 (11%). The absence of chest tightness was among 64 (87.7%) and present among nine (12.3%). The presence of seasonal variations was found among 28 (38.4%) and absent among 45 (61.6%). All other baseline characteristics have been highlighted in Table 1.

**Table 1:** Baseline characteristics of the study population

Parameters		Frequency (n=73)	Percentage (%)
Age	30- 39 years	9	12.32
	40-49 years	23	31.50
	50-59 years	29	39.72
	60-69 years	11	15.06
	70-79 years	1	1.36
Gender	Female	40	54.8
	Male	33	45.2
Asthma	Present	41	56
	Female	27	36
	Male	14	18
Occupation	Businessmen	15	20.5
	Cook	4	5.5
	Electrician	2	2.7
	Engineer	6	8.2
	Farmer	5	6.8
	Housewife	34	46.6
	Tailor	2	2.7
	Teacher	5	6.8
Symptoms	Cough		
	Absent	44	60.3
	Present	29	39.7
	Expectoration		
	Absent	53	72.6
	Present	20	27.4
	Shortness of breath		
	Absent	65	89
	Present	8	11
	Wheezing		
	Absent	54	74
	Present	19	26
	Chest tightness		
	Absent	64	87.7
	Present	9	12.3
	Family history of allergy		
	Absent	48	65.8
	Present	25	34.2
	Seasonal variation		
	Absent	45	61.6
	Present	28	38.4
Asthma treatment	Dry powdered inhaler (DPI)	16	21.9
	Metered dose inhaler (MDI)	19	26
	Nil	38	52
Comorbidities	Hypertension		
	Absent	12	16.4
	Present	61	83.6
	Type 2 diabetes mellitus		
	Absent	28	38.3
	Present	45	61.6

The mean values of the quantitative variable of metabolic syndrome have been shown in Table 2.

**Table 2:** Mean values of the quantitative variables

	Mean (n=73)	Std. deviation
Waist-hip ratio	0.55	0.43
Body mass effect	29.65	2.64
Worsening of symptoms	0.90	0.99
Systolic blood pressure (in mm of Hg)	151.09	19.11
Diastolic blood pressure (in mm of Hg)	90.00	6.73
Fasting blood sugar (mg/dL)	56.57	40.28
Hemoglobin A1C (HbA1C)	6.02	0.62
Serum insulin	23.08	9.30
Triglycerides	70.24	67.77
Total whole blood counts (/dl)	3560.95	3991.08
Pre-forced expiratory volume in the first second (FEV1)	1.03	0.79
Pre-FEV1/forced vital capacity(FVC)	0.75	0.93
Post FEV1	0.89	1.12
Post FEV1/FVC	0.93	1.13

It has been inferred that a reduction in BMI and waist circumference can improve the spirometry indices and reduce asthma symptoms, as shown in Table 3.

**Table 3:** Correlation of BMI and waist circumference (WC) with asthma at baseline and after six months FEV1: forced expiratory volume in the first second; FVC: forced vital capacity

		BMI	WC
Worsening of symptoms	Pearson's correlation	0.079	-0.109
	p-value	0.508	0.359
	N	73	73
Pre-FEV1	Pearson's correlation	-0.066	-0.021
	p-value	0.580	0.863
	N	73	73
Pre-FEV1/FVC	Pearson's correlation	-0.024	-0.010
	p-value	0.839	0.932
	N	73	73
Post FEV1	Pearson's correlation	-0.032	-0.076
	p-value	0.788	0.524
	N	73	73
Post FEV1/FVC	Pearson's correlation	-0.029	-0.019
	p-value	0.805	0.871
	N	73	73
After six months: Pre-FEV1	Pearson's correlation	-0.044	0.095
	p-value	0.715	0.428
	N	73	73
After six months: Pre-FEV1/FVC	Pearson's correlation	-0.064	0.059
	p-value	0.596	0.627
	N	73	73
Post-post FEV1	Pearson's correlation	-0.070	0.093
	p-value	0.561	0.441
	N	73	73
Post-postFEV1/FVC	Pearson's correlation	-0.073	0.057
	p-value	0.548	0.640
	N	73	73
After six months: Worsening of symptoms	Pearson's correlation	0.060	-0.005
	p-value	0.620	0.966
	N	73	73

It has been inferred that with the control of blood sugar levels and hyperinsulinemia, there is improvement in the spirometry indices of asthma and a lesser rate of worsening of symptoms, as shown in Table 4.

**Table 4:** Correlation of insulin resistance with asthma at baseline and after six months FEV1: forced expiratory volume in the first second; FVC: forced vital capacity

		Fasting blood sugar	Serum insulin
Worsening of symptoms	Pearson's correlation	-0.176	-0.060
	p-value	0.136	0.616
	N	73	73
Pre-FEV1	Pearson's correlation	-0.068	-0.204
	p-value	0.568	0.083
	N	73	73
Pre-FEV1/FVC	Pearson's correlation	-0.126	-0.101
	p-value	0.287	0.394
	N	73	73
Post FEV1	Pearson's correlation	-0.122	-0.124
	p-value	0.302	0.295
	N	73	73
Post FEV1/FVC	Pearson's correlation	-0.122	-0.113
	p-value	0.304	0.340
	N	73	73
After six months: Pre-FEV1	Pearson's correlation	-0.040	-0.177
	p-value	0.742	0.141
	N	73	73
After six months: Pre-FEV1/FVC	Pearson's correlation	-0.076	-0.103
	p-value	0.529	0.393
	N	73	73
Post-post FEV1	Pearson's correlation	-0.038	-0.144
	p-value	0.754	0.232
	N	73	73
Post-post FEV1/FVC	Pearson's correlation	-0.065	-0.117
	p-value	0.592	0.332
	N	73	73
After six months: Worsening of symptoms	Pearson's correlation	-0.108	0.072
	p-value	0.372	0.552
	N	73	73

It has been inferred that with the control of hyperlipidemia, there is improvement in the spirometry indices of asthma and a lesser rate of worsening of symptoms, as shown in Table 5.

**Table 5:** Correlation of lipid profile with asthma at baseline and after six months FEV1: forced expiratory volume in the first second; FVC: forced vital capacity

		Triglycerides	High-density lipoproteins (HDL)	Low-density lipoproteins (LDL)
Worsening of symptoms	Pearson's correlation	-0.211	-0.185	0.175
	p-value	0.077	0.123	0.144
	N	73	73	73
Pre-FEV1	Pearson's correlation	-0.262	-0.231	0.168
	p-value	0.027	0.053	0.160
	N	73	73	73
Pre-FEV1/FVC	Pearson's correlation	-0.294	-0.287	0.238
	p-value	0.013	0.015	0.046
	N	73	73	73
Post FEV1	Pearson's correlation	-0.262	-0.243	0.177
	p-value	0.028	0.041	0.139
	N	73	73	73
Post FEV1/FVC	Pearson's correlation	-0.291	-0.279	0.231
	p-value	0.014	0.018	0.053
	N	73	73	73
After six months: Pre-FEV1	Pearson's correlation	-0.399	-0.237	0.108
	p-value	0.000	0.044	0.363
	N	73	73	73
After six months: Pre-FEV1/FVC	Pearson's correlation	-0.410	-0.272	0.151
	p-value	0.000	0.020	0.202
	N	73	73	73
Post-post FEV1	Pearson's correlation	-0.377	-0.282	0.168
	p-value	0.001	0.016	0.155
	N	73	73	73
Post-post FEV1/FVC	Pearson's correlation	-0.402	-0.265	0.142
	p-value	0.000	0.024	0.230
	N	73	73	73
After six months: Worsening of symptoms	Pearson's correlation	-0.155	-0.234	0.192
	p-value	0.191	0.046	0.104
	N	73	73	73

It has been inferred that with the control of blood pressure, there is improvement in the spirometry indices of asthma and a lesser rate of worsening of symptoms, as shown in Table 6.

**Table 6:** Correlation of blood pressure with asthma improvement at baseline and after six months FEV1: forced expiratory volume in the first second; FVC: forced vital capacity

		Systolic blood pressure	Diastolic blood pressure
Worsening of symptoms	Pearson's correlation	-0.237	0.011
	p-value	0.044	0.924
	N	73	73
Pre-FEV1*	Pearson's correlation	-0.323	0.040
	p-value	0.005	0.735
	N	73	73
Pre-FEV1/FVC**	Pearson's correlation	-0.270	0.081
	p-value	0.021	0.495
	N	73	73
Post FEV1	Pearson's correlation	-0.298	0.041
	p-value	0.010	0.733
	N	73	73
Post FEV1/FVC	Pearson's correlation	-0.273	0.080
	p-value	0.019	0.499
	N	73	73
After six months: Pre-FEV1	Pearson's correlation	-0.237	0.120
	p-value	0.047	0.321
	N	73	73
After six months: Pre-FEV1/FVC	Pearson's correlation	-0.189	0.120
	p-value	0.115	0.319
	N	73	73
Post-post FEV1	Pearson's correlation	-0.241	0.121
	p-value	0.043	0.316
	N	73	73
Post-post FEV1/FVC	Pearson's correlation	-0.194	0.116
	p-value	0.105	0.335
	N	73	73
After six months: Worsening of symptoms	Pearson's correlation	-0.218	-0.010
	p-value	0.068	0.937
	N	73	73

## Discussion

This study aimed to assess the distribution and association of asthma among patients diagnosed with metabolic syndrome. The study included patients with a diagnosis of metabolic syndrome, and clinical factors such as body mass index, lipid profile, insulin resistance, and blood pressure were recorded. By evaluating the spirometry indices of all research participants, it was also possible to determine the relationship between these clinical indicators and asthma. Through this study, the relationship between control in the clinical markers of metabolic syndrome such as blood pressure, insulin resistance, hyperlipidemia, and body mass index and their effect on asthma was evaluated. The study included a sizable number of housewives as well. This occupational diversification might be viewed as a strength. Due to their lack of health knowledge and education, people from low socioeconomic backgrounds often struggle to control certain metabolic disorders. By focusing on a more diversified population that includes people from all social backgrounds, the likelihood of errors is therefore likely to be reduced.

As the majority of studies have focused on the metabolic dysfunction in asthma, inflammation is a frequent component [17]. Chronic airway inflammation characterizes the diverse condition known as asthma. Inflammation is not just limited to the airways; through inflammatory mediators, there is also significant distant cross-communication with other organs [17]. Adipose tissue raises the level of pro-inflammatory cytokines and causes systemic inflammation in asthma caused by obesity. A study on animals found that mice fed a hypercaloric diet acquired airway hyperreactivity that was dependent on NLRP3 but unrelated to adaptive immunity (just as it appears to regulate type 2 diabetes) [17]. To determine their relationship with asthma, the roles of diabetes, obesity, hypertension, and dyslipidemia were all taken into consideration.

A recent study by Huang et al. found that the median age of those suffering from metabolic syndrome was 54 years old, indicating that middle-aged people are more likely to develop the condition [22]. Similar to the previous study, more middle-aged patients in this one had metabolic syndrome.

In the current study, there were more females than males. According to research done by Pignataro et al., this can be attributed to female patients having a higher chance of developing asthma and metabolic syndrome [15]. Additionally, as this was a hospital-based study, there would have been more females there, which may have boosted the number of females in the study.

Through evaluation of cough, wheezing, shortness of breath, chest tightness, and spirometry, PFT reversibility was assessed, and asthma was identified in study participants. The likelihood of overlooking an asthma patient in the study was extremely low because all the factors related to asthma were discovered in the current investigation. The majority of study participants lacked symptoms such as coughing, wheezing, and shortness of breath, which was surprising. However, it should be mentioned that a sizable portion of individuals did wheeze, which suggests that asthma may have been present in some of them.

The majority of the study participants had a family history of allergen dispersion, showing that one of the key risk factors for asthma is family history. These findings were discovered to be consistent with research by Pignataro et al., who demonstrated a higher prevalence of asthma in women [15]. Additionally, it was discovered that the majority of the study participants suffered from asthma owing to seasonal changes, which further suggests that seasonal changes may also cause asthma [16,17]. 

The study participants' average type II diabetes prevalence was about 61%, and their average blood pressure was about 83%, demonstrating the relationship between metabolic syndrome and asthma. This was discovered to be comparable to other research that established the relationship between asthma and metabolic problems [11,18]. As previously mentioned, insulin resistance impairs the energy production in the mitochondria, which reduces oxygen supply and causes asthma by increasing bronchial reactivity through inhibition of the presynaptic M2 muscarinic receptor and being linked to skeletal muscle weakness, including the respiratory system. Insulin resistance also causes abnormal fat metabolism in the muscle and reduces glucose utilization [23,24]. Similar findings were also obtained through a study conducted by Assaad et al., where the prevalence of metabolic syndrome was reported in patients diagnosed with asthma [19].

In the current investigation, spirometric indices were also measured and represented as forced expiratory volume. The majority of study participants had diabetes and hypertension; hence, the finding that asthma was linked to metabolic syndrome can be further supported by the fact that spirometric indices provide quantifiable, objective assessments.

In the first and sixth months of the trial, the average BMI of all participants decreased significantly. The waist-hip ratio was also significantly lower during the follow-ups, indicating that research participants' risk of obesity had decreased. In addition to a positive reflection in insulin sensitivity indicating good control of diabetes among study participants, the level of HbA1C showed a reduction from 6.1 to 5.9 at the first follow-up. This showed a further decrease at the sixth-month follow-up. This was found to be extremely statistically significant.

Participants in the study had their blood pressure kept within normal ranges. The participants in the study had very low HDL levels and high triglyceride levels, which improved during follow-up visits and eliminated the risk of patients developing dyslipidemia. This finding supports the conclusion that effective cholesterol management improves spirometric indices. Additionally discovered to be statistically significant was this improvement in values. After metabolic syndrome was controlled, the FEV1/FVC ratio increased by 38% and 37% in the third and sixth months of follow-up, respectively. In follow-ups, it was also discovered that the deterioration of asthma symptoms was fewer. This may be linked to medical care and the regulation of metabolic parameters.

The results show that controlling diabetes, hypertension, obesity, and triglyceride values improved asthmatic symptoms, and this was determined to be statistically highly significant. These findings were discovered to be consistent with some research by Brumpton et al. that linked the occurrence of asthma with metabolic syndrome [14]. The study's findings were also discovered to be consistent with those of Camargo et al., who demonstrated that a reduction in BMI can lessen asthma symptoms [21]. Only a few researchers have examined the relationship between metabolic syndrome, chronic obstructive pulmonary disease (COPD), and asthma [25,26].

Strengths

Although there aren't many studies that specifically link each metabolic disorder (such as elevated body mass index, dyslipidemia, impaired insulin sensitivity, and hypertension) to asthma, this is one of the first to evaluate the metabolic syndrome as a whole (which includes all of the aforementioned metabolic disorders) and asthma. As patients of various ages were included in this study, it was able to determine the true link between metabolic syndrome and asthma as well as rule out any potential confounding effects of age in both asthma and metabolic syndrome. Because the data were gathered from a tertiary care institution, there will be no cluster effect and a wider range of participant distribution. Additionally, because this study involved the monitoring of numerous illness markers, a tertiary care hospital was the optimal location for the investigation. To demonstrate the link between asthma and metabolic syndrome, this study is one of the first to use a longitudinal study design with follow-ups. The fact that follow-ups continued for another six months can be seen as a strength since, after six months of monitoring metabolic markers such as blood sugar, blood pressure, and lipid profile, the precise relationship between each parameter and asthma was discovered.

Limitations

Since there was no control group in this study, it was unable to compare the results to the general population. However, the goal of the study was to determine whether asthma would improve with the care of diabetes, hypertension, lipid profiles, etc.; therefore, the inclusion of a control group did not bring any new information to the study. There was no random selection of the samples. An intentional sampling strategy was used. Additionally, because there were numerous ways for participants to achieve the inclusion requirements, random sampling would not have been practicable for this study. However, additional research planned in a specialized division like pulmonology with randomization can eliminate the confounders and improve the study's generalizability. This study is subject to selection bias as all participants were selected from the hospital. Although the study used a sufficient sample size, additional research with a larger sample size will be required to support the findings.

Implications

According to current investigations, there is a reduction in asthma symptoms with the control of metabolic disturbances. Thus, dietary changes along with an active lifestyle are to be prioritized.

Future scope

Future research can be suggested to determine the relationship between asthma and those with metabolic syndrome. To support the evidence provided by this investigation, randomized controlled trials can be performed.

Recommendations

It is imperative for the government to diagnose asthma among people with metabolic syndrome. If diagnosed at an early stage, asthma can be controlled. Hence, governmental organizations must include free diagnosis centers for asthma at primary health care and health and wellness centers. However, future research is necessary in this field to bring about an articulate finding.

## Conclusions

The results of the current study demonstrated that the regulation and maintenance of metabolic parameters such as body mass index, diabetes, hyperlipidemia, and hypertension aid in improving asthma control. All this evidence leads to the need for more research in this area, with a focus on the role of environmental factors in the development of asthma and metabolic dysfunction. There is unquestionably a need for personalized therapeutic strategies that include lifestyle modifications such as improving nutrition, regular exercising, and weight reduction for illness prevention, along with pharmaceutical therapies to improve the quality of life.
